# Developmental diversity in free-living flatworms

**DOI:** 10.1186/2041-9139-3-7

**Published:** 2012-03-19

**Authors:** José María Martín-Durán, Bernhard Egger

**Affiliations:** 1 Sars International Centre for Marine Molecular Biology, University of Bergen, Thormøhlensgate 55, 5008 Bergen, Norway; 2 Departament de Genética, Universitat de Barcelona, Avda. Diagonal 643, E-08028 Barcelona, Spain; 3 Department of Genetics, Evolution and Environment, University College London, Darwin Building, Gower St, London WC1E 6BT, UK; 4 University of Innsbruck, Institute of Zoology, Technikerstr. 25, 6020 Innsbruck, Austria

**Keywords:** Spiral cleavage, Hull cells, Blastomerenanarchie, Gastrulation, Phylotypic stage, Juveniles, Larvae

## Abstract

Flatworm embryology has attracted attention since the early beginnings of comparative evolutionary biology. Considered for a long time the most basal bilaterians, the Platyhelminthes (excluding Acoelomorpha) are now robustly placed within the Spiralia. Despite having lost their relevance to explain the transition from radially to bilaterally symmetrical animals, the study of flatworm embryology is still of great importance to understand the diversification of bilaterians and of developmental mechanisms. Flatworms are acoelomate organisms generally with a simple centralized nervous system, a blind gut, and lacking a circulatory organ, a skeleton and a respiratory system other than the epidermis. Regeneration and asexual reproduction, based on a totipotent neoblast stem cell system, are broadly present among different groups of flatworms. While some more basally branching groups - such as polyclad flatworms - retain the ancestral quartet spiral cleavage pattern, most flatworms have significantly diverged from this pattern and exhibit unique strategies to specify the common adult body plan. Most free-living flatworms (i.e. Platyhelminthes excluding the parasitic Neodermata) are directly developing, whereas in polyclads, also indirect developers with an intermediate free-living larval stage and subsequent metamorphosis are found. A comparative study of developmental diversity may help understanding major questions in evolutionary biology, such as the evolution of cleavage patterns, gastrulation and axial specification, the evolution of larval types, and the diversification and specialization of organ systems. In this review, we present a thorough overview of the embryonic development of the different groups of free-living (turbellarian) platyhelminths, including the Catenulida, Macrostomorpha, Polycladida, Lecithoepitheliata, Proseriata, Bothrioplanida, Rhabdocoela, Fecampiida, Prolecithophora and Tricladida, and discuss their main features under a consensus phylogeny of the phylum.

## Review

### Introduction

Flatworms (Platyhelminthes) are acoelomate, usually hermaphroditic, egg-laying bilaterians with multiciliated epithelial cells and are lacking a circulatory system, an anus and respiratory organs other than the epidermis [1]. The taxon is comprised of free-living and parasitic species, including flukes and tapeworms [2].

Since long, the embryonic development of flatworms has attracted attention of embryologists and phylogeneticists alike for their assumed central position in the evolution of the Bilateria or even the Metazoa [3]. Several hypotheses have been formulated to reconstruct the transition from ciliates to acoels [4,5], from cnidarian planula larvae to acoels [6] or from ctenophores to polyclads [7], and phylogenetic relationships were explored and discussed by studying the ontogeny of flatworms [3]. Today, the affiliation of the Platyhelminthes to the Spiralia (or Lophotrochozoa), especially apparent in polyclad flatworms, is widely accepted and the problematic position of acoels and nemertodermatids, traditionally regarded as members of the Platyhelminthes (see [8] and literature therein), is now commonly seen outside this phylum, either as sister group to all other bilaterians [9], as sister group of the Gnathostomulida [10] or as members of the deuterostomes [11].

**Figure 1:**
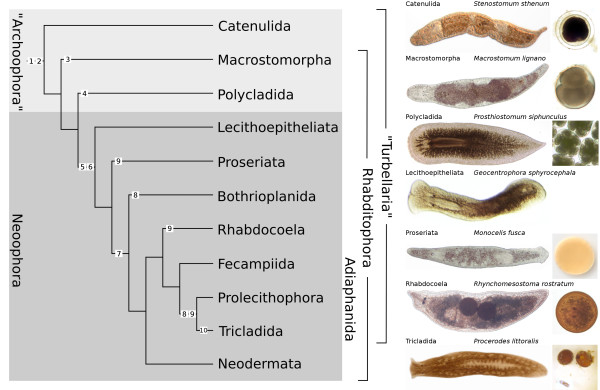
**Diversity of body plans and phylogeny of free-living platyhelminthes**. Left side, consensus tree of various published phylogenetic reconstructions. The Catenulida is the sister group of the Rhabditophora [15-18], the Macrostomorpha the sister group to all other Rhabditophora [2,15,19]. The Polycladida is the sister group to the Neoophora [2], and the Lecithoepitheliata is sister group to all other Neoophora [2,20]. Subsequently, the Proseriata is the sister group of all other Neoophora except the Lecithoepitheliata [16,20,21], while the Neodermata is sister group to Rhabdocoela and Adiaphanida (Fecampiida, Prolecithophora, Tricladida) [15,16,20,21]. The Rhabdocoela is sister group to Adiaphanida [15-17,20,21]. Within the Adiaphanida, Fecampiida is sister group to Prolecithophora plus Tricladida [15-17,20,21]. According to Willems and coworkers, the Bothrioplanida is sister group to Adiaphanida plus Neodermata, although their overall tree topology is different than depicted here, where Bothrioplanida is sister group to the Eulecithophora [22]. **1 **entolecithal eggs, **2 **quartet spiral cleavage, **3 **hull cells made from macromeres, **4 **larvae, **5 **ectolecithal eggs, **6 **hull cells made from micromeres, **7 **loss of spiral cleavage, **8 **disperse cleavage (*Blastomerenanarchie*), **9 **hull cells made of yolk cells (at least in some representatives), **10 **embryonic pharynx and "yolk larvae". Right side, live images of adult representatives of the major taxa of free-living flatworms with their developing embryos. *Stenostomum sthenum *with two developing zooids, fresh water, about 1 mm long. Mid-stage embryo, about 150 μm in diameter. *Macrostomum lignano*, marine, about 1 mm long. 4-cell stage, about 150 μm in diameter. *Prosthiostomum siphunculus*, marine, about 1 cm long. Several embryos per cocoon, several cocoons per egg plate. Embryos about 130 μm in diameter. *Geocentrophora sphyrocephala*, fresh water, about 1 mm long. No embryonic stage provided. *Monocelis fusca*, marine, about 1.2 mm long. Egg capsule of an unidentified proseriate, about 150 μm in diameter. *Rhynchomesostoma rostratum*, fresh water, about 1.3 mm long. Egg capsule with late embryo (note eyes) of a summer egg, about 170 μm in diameter. *Procerodes littoralis*, marine, about 4 mm long. 2 cocoons, the one to the right opened. Cocoon about 1 mm in diameter. Lower left corner hatched juvenile. Anterior of adult specimens to the left.

Traditionally, two broad classifications were used to subdivide the Platyhelminthes. According to their lifestyle, flatworms were classified either in free-living forms (former class "Turbellaria") including some parasitic groups such as the Fecampiida, and in strictly parasitic organisms (Neodermata, Figure 1). Here, we use the term "free-living flatworms" in the turbellarian sense, i.e., to encompass all flatworms other than the Neodermata. In addition, the structure of the oocyte was used as a systematic criterion. Flatworms with entolecithal eggs - eggs that contain all yolk needed for development - are called "Archoophora", and this condition is considered primitive or plesiomorphic, while the ectolecithy of the Neoophora (all other platyhelminth taxa, including the Neodermata) requires the invention of specialized yolk cell-producing organs, the vitellaria. Besides the oocyte, ectolecithal eggs also incorporate extra-embryonic yolk cells within the egg capsule [2,3,12-14].

Cladistic phylogenies of the phylum were established by Karling [23], Ax [14], Ehlers [2] and Smith et al. [24] based on morphological characters. The three latter phylogenies already display the "Turbellaria" and the "Archoophora" to be paraphyletic by not encompassing all descendants of the same common ancestor, the Neodermata and the Neoophora, respectively, the monophyly of which is supported. In addition, they established some internal affinities that have been accepted until recently, such as the group Seriata, comprising of the Tricladida and Proseriata, or the Rhabdocoela, which previously included also the Neodermata. In all these phylogenies, the Catenulida are regarded as the sister group to either only the Rhabditophora, or the Rhabditophora plus Acoelomorpha.

The use of molecular data and more elaborate cladistic techniques confirmed the overall picture proposed by earlier studies, namely the paraphyly of "Turbellaria" and "Archoophora" and the monophyly of Neoophora and Neodermata, but showed that the relationships among particular groups of flatworms are in fact more complex. Although some questions remain unanswered, e.g. the relationship between the Macrostomorpha, the Polycladida and the Lecithoepitheliata and their relationship to the remaining Rhabditophora (as to which is the most basally branching taxon), progress has been made towards defining a consensus internal tree of the phylum (Figure 1, see also [15]). The Acoelomorpha are no longer part of the Platyhelminthes proper, and Catenulida, Macrostomorpha and Polycladida are, possibly in this order, the most basally branching groups of flatworms, all of them showing entolecithal eggs. The Neoophora is comprised by the Lecithoepitheliata, the Proseriata, the Bothrioplanida, the Rhabdocoela, the Fecampiida, the Prolecithophora, the Tricladida and the Neodermata. The Seriata (Proseriata plus Tricladida) is no longer supported, and instead, the Tricladida, the Prolecithophora and the Fecampiida are now together in a monophyletic group called Adiaphanida [25] (Figure 1). Similarly, the Rhabdocoela has experienced severe rearrangements [22], and Neodermata has become a separate group. The position of the Bothrioplanida (previously considered to be proseriates [26]), however, is still not unambiguously resolved, as is the exact nature of the relationship between the Neodermata, the Adiaphanida, the Rhabdocoela and the Proseriata, or the relationship between the taxa within the Adiaphanida [16,22,25].

In a seminal work, the embryonic development of free-living flatworms known at the time was summarized and discussed by Bresslau [27], in particular comparing the "duet spiral cleavage" of acoels and the quartet spiral cleavage of polyclads with the more convoluted and unique development of rhabdocoels, triclads, *Bothrioplana *and *Fecampia*. While polyclads follow a relatively stereotypical spiral cleavage pattern, in triclads and other neoophorans spiral cleavage was found to be replaced by a seemingly irregular disperse cleavage, referred to as "*Blastomerenanarchie*". Quartet spiral cleavage was determined to be the most likely plesiomorphic cleavage pattern in platyhelminths, and four different types of development were proposed for the Neoophora, depending on their specific mode of encompassing the extra-embryonic yolk cells [3]. Later, the view that all Neoophora undergo an irregular cleavage pattern was changed by Giesa and Reisinger and coworkers [28,29]. They showed that neoophoran lecithoepitheliates and proseriates exhibit quartet spiral cleavage patterns despite the presence of extra-embryonic yolk cells within the egg, suggesting a gradual move away from spiral cleavage within the Neoophora [26,29]. They argue that several neoophoran taxa have originated independently from different archoophoran ancestors, explaining their different modes of engulfing the extra-embryonic yolk cells. The origin and formation of these so-called "hull cells" and its possible homology with the epibolic gastrulation of polyclads were considered central in reconstructing the evolution of developmental patterns in flatworms, as well as the nature of egg shell granules [30]. To date, the last comparative work on the embryonic development of free-living flatworms, still including the Acoelomorpha, was given by Baguñà and Boyer, with discussions on body axes formation and gastrulation [31].

Our current knowledge of flatworm ontogeny reveals a fascinating diversity that contrasts with the relative similarity of adult body plans observed among free-living platyhelminthes (Figure 1). Nowadays, molecular techniques have complemented more classical embryological approaches, putting some representative species on the level of other emerging invertebrate model systems. Herein, we review the existing literature dealing with the embryology of free-living Platyhelminthes *sensu stricto*, comprising the Catenulida and the Rhabditophora [17], under a consensus phylogeny. By doing so, we aim to create the adequate comparative framework in which testable hypotheses regarding the evolution and diversification of developmental modes in this phylum can be established.

### Embryogenesis of free-living flatworms

In this part of the review, we provide the main findings on embryonic development in each of the main taxa of free-living flatworms, with particular focus on early development (i.e. cleavage, cell lineage, gastrulation, establishment of axial identities, and presence of an intermediate stage). Table 1 summarizes the most important known embryonic traits of each group and allows a direct comparison among them.

**Table 1:** Comparison of the embryonic development of the main free-living flatworms

				Neoophora						
								Adiaphanida		
	Catenulida	Macrostomorpha	Polycladida	Lecithoepitheliata	Proseriata	Bothrioplanida	Rhabdocoela	Fecampiida	Prolecithophora	Tricladida
Egg	Entolecithal	Entolecithal	Entolecithal	Ectolecithal	Ectolecithal	Ectolecithal	Ectolecithal	Ectolecithal	Ectolecithal	Ectolecithal
Cleavage	Spiral (early)	Spiral (early)	Spiral	Spiral	Spiral (early)	Disperse	Irregular	Irregular	Disperse	Disperse
Specification^a^	?	Emb. blast.	Mosaic	Emb. blast.	Emb. blast.	Emb. blast.	Emb. blast.	Emb. blast.	Emb. blast.	Emb. blast.
Mesentoblast	?	-	4d^2^	4d	-	?	-	?	-	-
Gastrulation	?	Inverse epiboly (kind of)	Epiboly	Epiboly	Modified epiboly	-	Epiboly (kind of)	"Invagination"	Inverse epiboly (some)	-
Hull membrane^b^	?	Yes	-	Yes	Yes	Yes	Variable	-	Variable	Yes
Blastopore	?	?	Yes	Yes	Yes	-	-	-	-	-
Emb. Pharynx^c^	?	-	-	-	-	-	-	-	-	Yes
AP axis^d^	?	An-Veg	modified An-Veg	?	?	?	?	?	?	?
DV axis^e^	?	?	AB quad. ventral, CD quad. dorsal	An-Veg	An-Veg	?	?	?	?	?
LR axis^f^	?	?	BC quad. right, AD quad. left	?	?	?	?	?	?	?
Larva	Juvenile described as "larva"	-	Yes	-	-	-	-	Juvenile described as "larva"	-	Embryo described as "larva"

^a^Cell-type specification: in the Polycladida development is determinative. In the rest of the groups, organs develop from an embryonic blastema (Emb. blast.)

^b^Origin of *hull cells*: Macrostomorpha, blastomeres 2A-2D; Lecithoepitheliata and some Proseriata, animal micromeres; some Rhabdocoela and *Bothrioplana*, unspecified micromeres or blastomeres; Tricladida, some Prolecithophora, some Rhabdocoela, some Proseriata, yolk cells

^c^Embryonic pharynx

^d, e, f^Anteroposterior, dorsoventral and left-right axis, respectively. An-Veg means animal-vegetal axis and quad. means quadrant

#### Archoophora

The "Archoophora" (Catenulida, Macrostomorpha and Polycladida) is a paraphyletic group encompassing all flatworms with entolecithal eggs. In entolecithal eggs, all yolk is contained within the oocytes. Archoophorans exhibit quartet spiral cleavage, at least during the early zygotic divisions. Knowledge of embryonic development in catenulids is scarce. In the Macrostomorpha, development diverges from the 8-cell stage, with the formation of an external yolk mantle from the four vegetal yolky macromeres that eventually cover the embryo and will be later resorbed. The juvenile thereby develops from the inner mass of cells, which is organized into an embryonic blastema. The Polycladida retain the normal quartet spiral cleavage, although some differences with other spiralian phyla (e.g. annelids, molluscs or nemerteans) are observed. Their development is determinative and gastrulation occurs through epiboly of the animal micromeres over the vegetal cells. Some species of this group feature an intermediate larval phase.

#### Catenulida

Catenulids are predominantly freshwater animals in the millimeter range with only about 100 described species worldwide, about half of which are belonging to the genus *Stenostomum *[32]. Data about the embryonic development of catenulid species are few and far between. Observations on the embryonic development of the group have been undertaken with species of the genus *Stenostomum*, namely *S. leucops *and *S. sthenum *[33,34] and with *Catenula lemnae *[35]. Single oocytes are covered by either a thick [33] or thin [35] egg shell.

Both in *C. lemnae *and in the two *Stenostomum *species, the embryonic development is described as spiralian at least in the early cleavage stages [34,35]. The first cleavage divisions can occur when the egg is still residing in the parent. At the 4-cell stage, the blastomere D divides first, so that a temporary 5-cell stage can be observed, before the 8- and then the 16-cell stage are reached, which are described to look similar to polyclads of the same cell stage [35]. In *S. leucops*, the development proceeds slowly and takes several days to reach the 8-cell stage, at which point it remains in a diapause for two more months, after which a thin transparent layer surrounds the embryos [33]. In *S. sthenum*, on the other hand, the diapause starts after five or maximal twenty blastomeres have appeared. At temperatures of 17-20°C, the first cleavage is noted after about one hour, and the 4-cell stage is reached after two hours [34]. At the 2-cell stage, there is no marked size difference between the blastomeres, except when parts of a blastomere are being extruded into the periembryonic liquid [34]. Later embryonic stages following the diapause are not described yet for any catenulid.

*S. sthenum *hatches as a directly developed juvenile, also called "archaezooid" [34]. Interestingly, for *Rhynchoscolex simplex*, a so-called Luther's larva was described. While the embryonic development and the hatching could not be observed, in spring these larvae were found at the same location as later the adults. The observed larvae are very slender with 30 μm in width and 800 μm in length, and are very similar to the adults, the only differences being the presence of a statocyst in the larvae, which is lost during further development, and slightly longer (4.5 μm instead of 3-4 μm in adults) cilia in the head region [36].

#### Macrostomorpha

**Figure 2:**
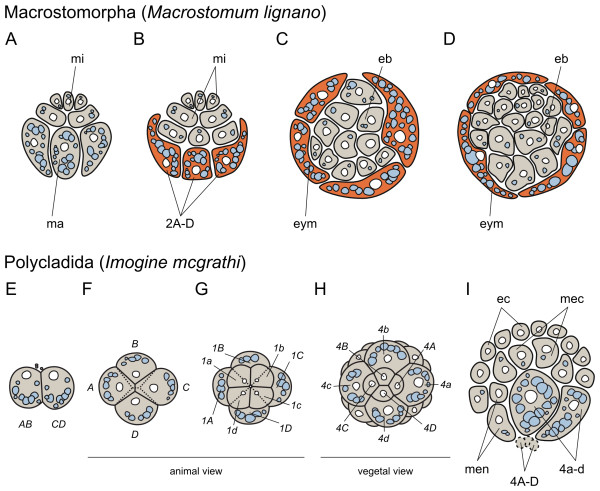
**Summary of the embryonic development of Macrostomorpha and Polycladida**. (A-I), schematic representations of the early macrostomid (modified from [43]) and polyclad development (adapted from [45,46]). In macrostomids, early cleavage follows the typical quartet spiral cleavage pattern (A) up to the 8-cell stage, after which the four vegetal macromeres 2A-2D flatten (B) and form a yolk mantle that covers the embryo (C-D) that will be eventually replaced by the definitive epidermis. The rest of the blastomeres remain in the inner region and form an embryonic blastema from which the organs of the juvenile develop. Polyclads, on the contrary, exhibit a quite conserved quartet spiral mode of development (E-I), except that macromeres 4A-4D are smaller than the micromeres 4a-4d (H). Gastrulation occurs through epiboly of the animal micromeres over the vegetal macromeres (I). As a peculiarity of polyclad development, the macromeres 4A-4D (represented with a slashed line in H) and the micromeres 4a-4c degenerate, and thereby, the whole endoderm and a large part of the mesoderm is originated by the 4d micromere. In all schemes, an idealized animal-vegetal axis cross section of the embryo is represented (animal to the top, vegetal to the bottom), unless otherwise indicated. Yolk granules are colored in light blue, hull cells in orange and embryonic cells in gray. Drawings are not to scale. *eb *embryonic blastema, *ec *ectoderm, *eym *embryonic yolk mantle, *ma *macromere, *mec *mesoectoderm, *men *mesoendoderm, *mi *micromere.

The Macrostomorpha comprise small flatworms in the two taxa Haplopharyngida (consisting of 2 marine species) and Macrostomida (about 230 marine and freshwater species [32]). To present, studies on the embryonic development are restricted to the Macrostomida, and, with one exception (*Microstomum lineare*, [35]), to the genus *Macrostomum*. Both single (e.g. *Macrostomum lignano*) and multiple (up to 20, e.g. *Macrostomum romanicum*) embryos per egg shell or cocoon are deposited. Three polar bodies are extruded at the animal pole, which later become incorporated into the embryo (*Macrostomum appendiculatum, M. lignano*). Large cytoplasmatic protuberances ("blebbing") occur predominantly in the undivided oocyte, but are also seen during cleavage [35,37,38]. At the two-cell stage, one blastomere (CD) is usually slightly larger than the other (AB), and by laeotropic (left-handed) division arrives at the four-cell-stage, where blastomere D is often, but not always largest. The third cleavage is dexiotropic and produces a quartet of micro- and macromeres (Figure 2A), the micromeres on the animal pole being almost equally large as the vegetal macromeres. The fourth cleavage is laeotropic again. The early cleavage pattern is of quartet spiral nature in macrostomids, as was shown in early accounts on *M. appendiculatum *and *Macrostomum viride *[39,40]. In *M. appendiculatum*, the existence of so-called hull cells has been described for the first time in the genus *Macrostomum *[41], and has later been confirmed to occur also in *M. romanicum *[42] and *M. lignano *[38,43]. Interestingly, in *M. viride *the embryo seemingly retains the spiral nature of cleavage up to the 128-cell stage and even the mesentoblast 4d is described, while no hull cells are mentioned [35]. In *Microstomum*, cleavage was only observed up to the 8-cell stage [35,44].

Hull cells are large, yolk-rich blastomeres of embryonic origin (macromeres 2A-2D, [38,41]) that start to flatten and surround the remaining blastomeres in the 16-cell stage (Figure 2B). With progressing development, the four hull cells do not divide anymore [38], but expand and flatten further, transforming into a thin yolk mantle (Figure 2C-D), which is eventually replaced by the definitive epidermis emerging from the mesenchymal space underneath [47] (for *Macrostomum hystricinum marinum*), and become incorporated into the gut [38]. The growth of the definitive epidermis initiates at the animal (anterior) pole and continues ventrally and then dorsally to cover the whole embryo [41]. Seilern-Aspang holds that the future orientation of the body axis is already visible in the two-cell stage, where an area in the center of the embryo has basophilic properties, which during development shifts to the side of the embryo, designated the future ventral side. The animal pole will become the anterior part, and the vegetal pole the posterior part of the animal. At the future ventral side of the animal, the organ primordia are developing [41,43]. Ciliogenesis of the multiciliary epidermis cells starts at about 50% developmental time in *M. hystricinum marinum, M. romanicum *and *M. lignano *[42,43,47].

While a typical gastrulation movement cannot be observed in *Macrostomum*, the covering of the embryo by hull cells was interpreted as a kind of "inverse epiboly", whereas the formation of the gut primordium by small blastomeres surrounding the inner yolk mass can be called an epibolic growth with the function of a late gastrulation, but not being homologous to gastrulation of other flatworms, e.g. polyclads [41,43]. The area around the closure of the definitive epidermis has been proposed as a blastopore [30].

Embryogenesis in *Macrostomum *concludes with the hatching of a directly developing juvenile after about 4-7 days.

#### Polycladida

This taxon consists of mostly large (centimeter range), almost exclusively marine animals and is divided into two suborders: the Cotylea (about 350 species) with a prominent sucker posterior of the female genital opening, and the Acotylea (about 450 species [32]) without such a sucker [48]. All studied representatives of the Polycladida show a quartet spiral cleavage reminiscent of annelids and molluscs [48-54]. To date, the most comprehensive report of the early embryonic development, up to about the 100-cell stage, was undertaken by Surface using both live observations and serial sections of fixed embryos of *Hoploplana inquilina *[51], followed by a microinjection-based study on the same species by Boyer and coworkers [55].

In polyclads, cell blebbing is common in early stages, especially the 1-cell stage [51,54]. The first two cleavages are meridional and are described as being equal or slightly unequal (Figure 2E-F). Of these four blastomeres, the largest (if recognizable) is designated as the D blastomere, giving eventually rise to the mesentoblast [51]. During the third cleavage, an animally situated micromere quartet is given off right-handedly (dexiotropically) of the vegetal macromere quartet (Figure 2G). At this stage, the macromeres are usually bigger than the micromeres (with some exceptions among stylochids, see [52,54]). Subsequent cleavages alternate between laeotropic and dexiotropic divisions, owing to the oblique angle of the mitotic spindles [51]. After the sixth cleavage division and after having given off four micromere quartets, the macromeres 4A-D are significantly smaller than the corresponding micromere quartet 4a-d [51,56] (Figure 2H-I). From this point onwards, the macromeres and the fourth quartet micromeres (except 4d) stop dividing and are eventually resorbed into the embryo [51,55].

The mesentoblast (usually blastomere 4d) is the stem cell of the mesodermal bands and also contributes to parts of the endoderm in spiralians [57]. In polyclads, micromere 4d is responsible for the origin of large parts of the meso- and the whole endoderm and forms bilateral mesodermal bands [51,55]. Nevertheless, cell lineage studies have revealed that in polyclads, the mesoderm is not only formed by the mesentoblast, but also by micromere 2b, which is forming circular musculature and also contributes to the ectoderm [51,55]. Additionally, there is conflicting evidence about the nature of the actual mesentoblast in polyclads: according to Kato [52], blastomere 4d is the mesentoblast, dividing horizontally into 4d^1 ^and 4d^2^, while Surface [51] and van den Biggelaar [57,58] hold that blastomere 4d first divides along the animal-vegetal (AV) axis into 4d^1 ^and 4d^2^, both of which then divide bilaterally (horizontally). The 4d descendant lying nearer to the animal pole contributes towards meso- and endoderm and is thus comparable to the 4d blastomere (the mesentoblast) in most annelids and molluscs, while the more vegetally located 4d descendant in polyclads is solely contributing to the endoderm [51,58]. Also, Surface [51] calls 4d the mesentoblast, while labeling its mesentoblastic descendant 4d^2 ^(following the spiralian nomenclature established by Conklin [59]) and its entoblastic descendant as 4d^1^, while van den Biggelaar [58] claims this is a mislabeling and swaps Surface's 4d^1 ^and 4d^2 ^labels, calling his 4d^1 ^the mesentoblast. Provided that the observations of Surface [51] and van denBiggelaar [57,58] are correct in that the micromere 4d first divides along the AV axis before its descendants divide horizontally, the mesentoblast in polyclads is 4d^2^, following the spiralian nomenclature established by Conklin [59].

A series of blastomere ablation studies by Boyer (summarized in [55]) showed that polyclad development is determinative and mosaic and indicated that the A quadrant of the blastomere quartet corresponds to left ventral, B to the right ventral, C to the right dorsal and D to the left dorsal side of the future larva [55]. Specification of the dorso-ventral (DV) axis is unlikely to occur before the 8-cell stage, as polyclads are equally or nearly equally cleaving spiralians. After formation of the fourth micromere quartet, blastomere 4b moves inside the embryo and gets in contact with the animal micromeres, suggesting that a similar mechanism for DV axis specification - induction by cell-cell contacts - is taking place in polyclads as in molluscs [55,57]. The DV axis becomes apparent with the bilateral division of the mesentoblast, its progeny defining the ventral side [45]. The anteroposterior (AP) axis is considered to be derived from the AV axis, declining to one side [45,52].

Gastrulation - leading to a stereogastrula - occurs via epiboly, starting from the animal pole and covering the embryo with ectoderm from all sides, leaving a soon to be closed blastopore at the vegetal pole [51,60,61]. The pharynx primordium invaginates at the vegetal pole of the animal from descendants of micromeres 2a, 2c and 3d [55]. Like the pharynx, the brain anlage is of ectodermal origin and stems from late first quartet micromeres 1a^112212^-1d^112212^, near the animal pole, but later shifts to a more anterior position, while the gut bends posteriorly [45,51,52].

**Figure 3:**
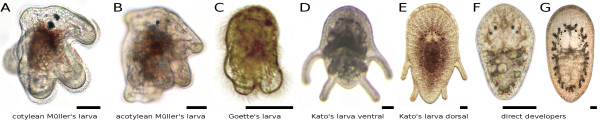
**Larval types and juveniles of Polycladida**. (A) Müller's larvae of a cotylean (*Prosthiostomum siphunculus*) and (B) an acotylean species (*Planocera multitentaculata*), both hatching with eight lobes and three eyes (two cerebral eyes and one epidermal eye). (C) Goette's larva of the acotylean *Imogine mediterranea*, hatching with four lobes and a cerebral and an epidermal eye. (D-E) Kato's larva of the acotylean *Planocera reticulata*, hatching with eight lobes and 12 eyes and being dorsoventrally flattened. (D) Ventral side with four lobes around the mouth visible, (E) from dorsal. (F) Directly developing juvenile of the acotylean *Pseudostylochus obscurus*, hatching with no lobes and four eyes. (G) Directly developing juvenile of an undetermined acotylean, hatching with no lobes and 12 eyes. All scale bars are 50 μm. Photograph (C) is courtesy of Mehrez Gammoudi.

Eventually, embryonic development gives rise to a juvenile or one of three larval types (Figure 3). Almost all cotylean and some acotylean polyclads feature an eight-lobed and three-eyed spherical Müller's larva. Only acotyleans show directly developing juveniles with four or 12 eyes, or a four-lobed and two-eyed Goette's larva or a dorso-ventrally flattened eight-lobed and 12-eyed Kato's larva [54,62,63]. The latter was previously described as an "intracapsular larvae" [52], but was later found to be predominantly hatching as a 12-eyed larva and not a metamorphosed juvenile [53,54]. Another case of an intracapsular larva was described, however, for the cotylean *Amakusaplana acroporae*, where a Müller's larva with 8 rudimentary lobes was found to metamorphose inside the egg shell and hatching as a mostly 9-eyed (8 cerebral and 1 epidermal eye) dorsoventrally flattened juvenile without lobes [64].

#### Neoophora

The Neoophora feature oogonia that are divided into a germarium and a vitellarium, producing oocytes and yolk cells, respectively. The egg contains both oocyte(s) and extra-embryonic yolk cells. Early development is very diverse across neoophoran groups. While some of them partially retain the quartet spiral cleavage (Lecithoepitheliata and Proseriata), others (Bothrioplanida, Rhabdocoela, Fecampiida, Prolecithophora and Tricladida) present divergent modes of cleavage, also with an impact on gastrulation. All of them have developed mechanisms to engulf the external yolk cells into the developing embryo, usually by forming one or more temporary epidermises, or hull membranes. Juveniles develop from an embryonic blastema with the ventral side facing outwards, as observed in macrostomids.

#### Lecithoepitheliata

**Figure 4:**
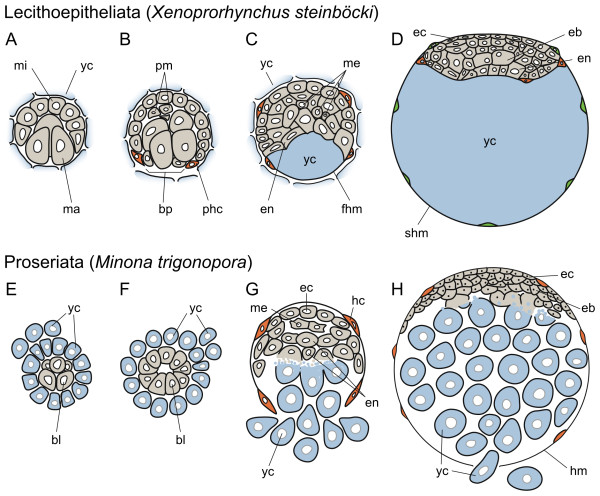
**Summary of the embryonic development of Lecithoepitheliata and Proseriata**. (A-H), schematic representations of the early development of lecithoepitheliates and proseriates (both modified from [29]). Lecithoepitheliates exhibit regular quartet spiral cleavage (A) and gastrulate by epiboly of the micromeres over the vegetal macromeres (B). During gastrulation, however, the micromeres 2a-2d and 3a-3d at the edge of the blastopore differentiate into hull cells, which engulf a portion of the yolk (in *X. steinöcki*, C) or the whole portion of maternally supplied vitellocytes (in *G. applanata*). The inner mass of blastomeres differentiates into an embryonic blastema that occupies the future ventral side of the embryo, and in *X. steinböcki *a second hull membrane is formed to incorporate the remaining yolk cells inside the eggshell (D). In proseriates, quartet spiral cleavage is only observed up to the 8-cell stage (E). After that, the embryo develops first into a coelogastrula (F) and later into a compact discoidal stereoblastula in which 6 peripheral blastomeres differentiate into a hull membrane that engulfs the yolk cells (G). As in lecithoepitheliates, the inner blastomeres form a discoidal embryonic blastema that occupies the future ventral side of the embryo (H). In all schemes, an idealized animal-vegetal axis (ventral-dorsal axis in D and H) cross section of the embryo is represented (animal/ventral to the top and vegetal/dorsal to the bottom). Yolk cells are colored in light blue, primary hull cells in orange, secondary hull cells in green and embryonic cells in gray. Drawings are not to scale. *bl *blastomere, *bp *blastopore, *eb *embryonic blastema, *ec *ectoderm, *en *endoderm, *fhm *first hull membrane, *hm *hull membrane, *ma *macromere, *me *mesoderm, *mi *micromere, *hc *hull cells, *phc *primary hull cells, *pm *primary mesoderm, *shm *second hull membrane, *yc *yolk cell.

This taxon is divided into the marine Gnosonesimidae (6 species) and the freshwater Prorhynchidae (about 30 species) [32]. Embryonic development has been described for the three prorhynchid species *Prorhynchus stagnalis *[65], *Xenoprorhynchus steinböcki *[29], and *Geocentrophora applanata *[66]. The embryo shows a typical unequal quartet spiral cleavage, with the D blastomere being somewhat larger than their sister cells [29]. In contrast to the situation observed in polyclads, the macromeres 4A-4D are big and originate the endoderm (Figure 4A). A true mesentoblast, blastomere 4d, is also observed (peculiarly, Reisinger and coworkers label blastomere 4D as the mesentoblast), from which the mesoderm in two bands is formed, as in other spiralians [29].

In *Xenoprorhynchus*, gastrulation consists in an epibolic movement of the animal micromeres over the vegetal macromeres at the 25-30 cell stage [29] (Figure 4A). Once it is completed (at about the 50-cell stage), ectodermal micromeres at the edge of the blastopore (micromeres 2a-2d and 3a-3d) flatten and differentiate into sheath epidermal cells, or a hull membrane (Figure 4B). This transitory epidermis covers the embryo and extends at the vegetal pole to engulf a small portion of the extra-embryonic yolk cells, which form a syncytium [29] (Figure 4C). The blastomeres at the animal pole absorb this yolk, proliferate and form an elongated and dorsoventrally flattened blastema that corresponds to the ventral side of the embryo. The first hull membrane is preserved only until the engulfed yolk cells have been absorbed by embryonic blastomeres and hull cells are then incorporated into a superficial layer of the embryo. Simultaneously, a second hull membrane (also of blastomere origin) differentiates, starting from the ventral (i.e., outer) side of the embryo and incorporates the majority of the extra-embryonic yolk cells, which occupy now the future dorsolateral region of the embryo (Figure 4D). Finally, the organs differentiate in the ventral embryonic blastema and the hull membrane is replaced by the definitive body wall epidermis [29]. It is not clear whether cells of the second hull membrane take a place in the final epidermis. Both brain and pharynx primordia are of ectodermal origin [29].

The embryonic development in *Geocentrophora *is in large parts similar to *Xenoprorhynchus*, but involves the formation of a coeloblastula. The primary hull membrane covers all yolk cells and, without the formation of a second hull membrane, is directly replaced with the definite epidermis, originating at the ventral side of the embryo. Different to *Xenoprorhynchus*, in *Geocentrophora *a contribution of the hull cells to the definite epidermis can be excluded [66]. Both in *Xenoprorhynchus *and *Geocentrophora*, the pharynx develops at the other side as the original blastopore was located. Finally, a directly developing juvenile hatches.

#### Proseriata

Proseriates contain marine and freshwater species and are classified into the Lithophora (about 400 species) and the Unguiphora (about 40 species) [32]. The embryonic development of proseriates has been studied in *Monocelis fusca *[28], *Minona trigonopora *[29] and *Otomesostoma auditivum *[26] (all Lithophora). A stereotypical quartet spiral cleavage is easily recognized up to the 8-cell stage, with equal or slightly unequal cell divisions, depending on the species (Figure 4E). After this point, cleavage diverges and no pattern is discerned. Early divisions lead to the formation of a coeloblastula (Figure 4F), and the appearance of "abortive blastomeres" [26,28,29]. These are formed after extremely unequal cell divisions in which one of the daughter cells receives almost no cytoplasm. Often peripherally located, their fate is uncertain, although most of them seem to perish [26]. As cleavage proceeds, the primary blastocoel disappears and a compact discoidal stereoblastula appears. The loss of spiral cleavage after the 8-cell stage hinders the identification of a true mesentoblast in proseriates. In *Monocelis*, some yolk cells build an epithelium around the yolk mass and the embryo within [28].

Simultaneous to the formation of a stereoblastula, the uptake of extra-embryonic yolk cells starts. Individual blastomeres of the periphery differentiate into sheath cells and form a 6-cell hull membrane that covers the embryo [26,28,29] (Figure 4G). The open area of the closing hull membrane has been proposed as the blastopore [26,28,29]. The two vegetal-most sheath cells start absorbing the extra-embryonic yolk cells, which are thus incorporated into the embryo. As in lecithoepitheliates, the incorporated yolk occupies a dorsal position, whereas the stereoblastula, now an elongated and dorsoventrally flattened blastema, is in the ventral side of the embryo (Figure 4H). In the latter, organogenesis takes place, first by the specification of an anterior head primordium and a posterior pharynx primordium, which eventually results in the definitive embryo. The hull membrane cells are replaced by the definitive body wall epidermis. Usually one juvenile hatches per egg capsule, but sometimes also two or more.

#### Bothrioplanida

**Figure 5:**
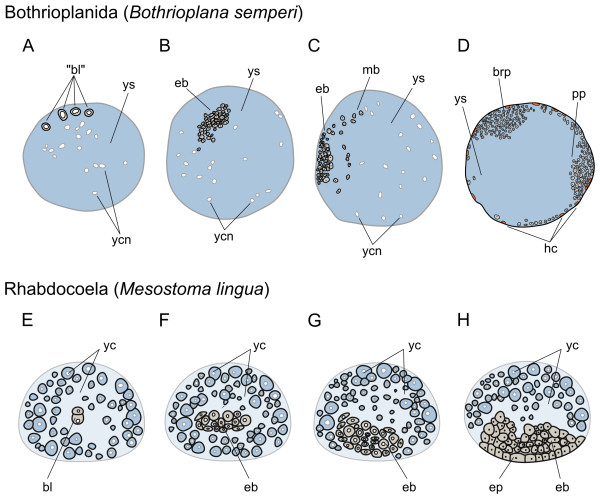
**Summary of the embryonic development of Bothrioplanida and Rhabdocoela**. (A-H), schematic representations of the early development of bothrioplanids (modified from [26]) and rhabdocoels (modified from [67]). *Bothrioplana *lays eggs containing two oocytes and many yolk cells, which are fusing to a yolk syncytium before the egg is laid. The oocytes undergo two meiotic divisions and give rise to 8 "blastomeres" (gametes) (A), which further divide to build an embryonic blastema (B). Migrating blastema cells (C) provide hull cells enveloping the yolk syncytium and the blastema cells, which are accumulating in the brain primordium and the pharynx primordium (D). In rhabdocoels, the first cell division is equatorial, giving rise to an animal micromere and a vegetal macromere (E). Proliferation of these two initial cells forms a discoidal embryonic blastema, which is first placed in the middle of the egg (F) and later moves to one side (G), which will become the future ventral side of the embryo. The epidermis differentiates from this embryonic blastema, as do the other organs, and engulfs the mass of external yolk cells (H). In all schemes, an idealized animal-vegetal axis (ventral-dorsal axis in D, G and H) cross section of the embryo is represented (animal/ventral to the top, vegetal/dorsal to the bottom in bothrioplanids and vegetal/ventral to the bottom, animal/dorsal to the top in rhabdocoels). Yolk cells are colored in light blue, hull cells in orange and embryonic cells in gray. Drawings are not to scale. *bl *blastomere, *"bl" *"blastomeres" which are gametes, *brp *brain primordium, *eb *embryonic blastema, *ep *epidermis, *hc *hull cells, *mb *migrating blastomeres, *pp *pharynx primordium, *yc *yolk cell, *ycn *yolk cell nuclei in a yolk syncytium *ys*.

Previously being considered close to the Tricladida ("Protriclades") [26] or the Proseriata [21], the current systematic position of the Bothrioplanida within the Neoophora is only preliminarily resolved (Figure 1). The embryonic development of its only described representative, *Bothrioplana semperi*, is characterized by "parthenogenetic octogametogenesis" [26], that is parthenogenesis starting from two primary oocytes per egg, resulting in 8 diploid "blastomeres", which are actually gametes (Figure 5A). *Bothrioplana *has reduced male organs and is obligatory parthenogenetic with little chromosomal variation worldwide [26].

The 8 "blastomeres" descendant from the primary oocytes disperse in a kind of *Blastomerenanarchie *and divide unequally, with no trace of spiral cleavage being discernible. The yolk cells have already merged to a syncytium enclosing the embryo in the center, which then moves to the periphery of the yolk syncytium forming an embryonic blastema (Figure 5B-C). Small blastomeres from the blastema start growing a hull membrane surrounding the whole yolk mass and the peripheral embryo [26,27] (Figure 5D). Later, the hull cells will be partially contributing to the epidermal layer. Three distinct parts of the embryonic blastema differentiate to anlagen for the brain, the pharynx and the genital apparatus, the latter developing slowest. The pharynx anlage, shifting caudally, defines the ventral side of the embryo. From both sides of the anlagen, a second hull membrane spreads to encompass the yolk mass once again. The second hull cells are probably not contributing to any definitive embryonic structures and are being replaced by the definitive epidermis emerging under the second hull membrane [26]. The juveniles hatch with a fully developed pharynx, but with only a rudimentary gut consisting of a hull layer separating the yolk in the gut lumen from the mesenchyme.

#### Rhabdocoela

This is a highly diverse and cosmopolitan group of flatworms including the Dalytyphloplanida (about 1000 species), the Endoaxonemata (1 described species), the Kalyptorhynchia with about 550 species and the parasitic or commensal Temnocephalida (circa 160 species) [32]. The embryonic development of several species (almost all Dalytyphloplanida) has been described, especially at the morphological level [67-76]. As in *Bothrioplana*, cleavage is not of the quartet spiral type, although one author states that the early cleavage (4- to 8-cell stages) of *Bresslauilla relicta, Paravortex *and *Phaenocera *hints at spiral cleavage [35]. The first cell division is unequal and occurs in the equatorial plane instead of along the AV axis, dividing the zygote into an animal micromere and a vegetal macromere (Figure 5E). Homology of these two cells to the blastomeres AB and CD of canonical spiralians has been proposed by Giesa [28]. Different to normal spiral cleavage, in eggs of *Mesostoma ehrenbergi *and *Bothromesostoma personatum*, after the 2-cell stage the macromere constricts two more micromeres, before the first division of a micromere occurs [68,72]. After the distinctive early divisions, cleavage proceeds without a defined pattern, giving rise to a compact and flattened or morula-like mass of irregular blastomeres at the center of the egg (Figure 5F).

Epiboly of animal micromeres to cover the vegetal blastomeres and part of the extra-embryonic yolk cells, as in other neoophoran groups with well-defined blastulas, does not occur. In the typhloplanid family Mesostomidae, some peculiar developmental features can be observed. Species of the genera *Mesostoma *and *Bothromesostoma *have been shown to produce seasonal eggs: small summer eggs with a thin transparent egg shell, that fully develop within the parent animal (ovovivipary), and bigger winter eggs with a thick, red-brown colored egg shell, that are developing outside of the parent animal. In summer eggs of *Mesostoma*, some yolk cells transform into sheath cells and form sort of a hull membrane that engulfs the rest of the yolk cells and the mass of blastomeres [67,68]. Interestingly, in winter eggs of the same *Mesostoma *species and in summer eggs of *Bothromesostoma*, such a hull membrane made of yolk cells is not being formed. In kalyptorhynch rhabdocoels, micromeres move through the yolk mass and form an embryonic epithelium around the yolk, after which blastomeres of endodermal origin act as vitellophages. Migration of blastomeres to the periphery is described as gastrulation [72], whereas in *Mesosostoma*, a morphogenetic process comparable to gastrulation in other animals is absent [67], as it was noted since the very first observations of their development [68]. In the dalyellioid *Paravortex gemellipara*, embryonic phagocytes are incorporating yolk cells before a hull membrane of embryonic origin is formed [71].

After an active phase of proliferation, the blastula, or embryonic blastema, moves to one side of the egg capsule and becomes the future ventral side, as observed in other neoophoran flatworms (Figure 5G). It elongates and acquires bilateral symmetry, and the organs start differentiating. First, the brain primordium on the future anterior pole and a bit posteriorly, the pharynx primordium emerge. In kalyptorhynchs, the embryonic epithelium differentiates into the definitive epidermis [72], whereas in *Mesostoma*, the epidermis differentiates in the most ventral side of the blastema and migrates peripherally towards the other side of the egg, engulfing the yolk cells, which occupy now the dorsal side of the embryo (Figure 5H). Thus, the differentiated epidermis is replacing the embryonic hull membrane made from yolk cells [67]. The gastrodermis differentiates in the inner side of the blastema and progressively absorbs the yolk cells. As organogenesis proceeds, the nervous system and the eyes form from the brain primordium, the pharynx connects with the exterior through the mouth and the posterior-most region differentiates into the caudal region and the reproductive system. The parenchyma and the musculature develop throughout the embryonic blastema [67,68]. Last, directly developing juveniles break through the egg shell.

#### Fecampiida

**Figure 6:**
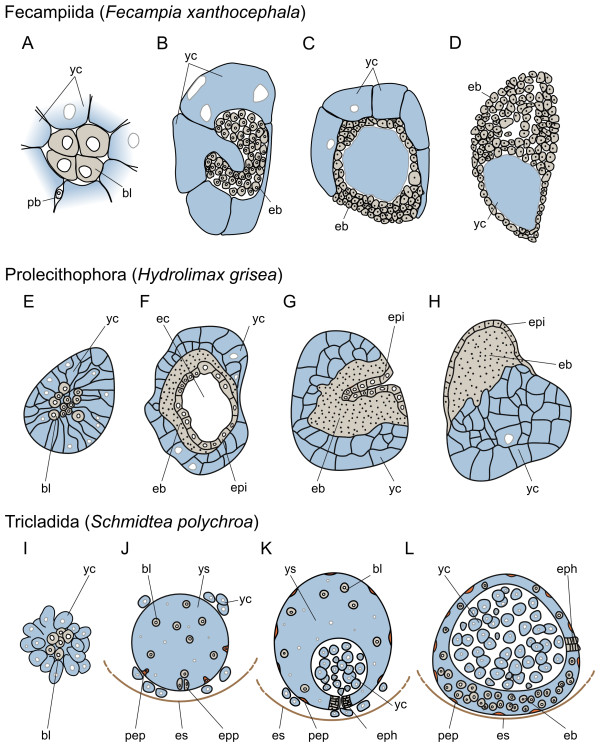
**Summary of the embryonic development of Adiaphanida**. (A-L), schematic representations of the early development of fecampiids (modified from [77]), prolecithophorans (modified from [79]) and triclads up to the incorporation of the external yolk cells by the embryo. In fecampiids, early cleavage seems not to be of the disperse type (A), as in the other adiaphanids. After cleavage, the embryo forms an open pouch (B) and incorporates inside this cavity part of the yolk cells (C). Subsequently, the embryo extends its walls to the periphery of the yolk mass, incorporating the remaining yolk and adopting a hemispherical shape (D). The yolk becomes restricted to the posterior part of the embryo, while the blastomeres in the opposite pole proliferate and form an embryonic blastema. In prolecithophorans, disperse cleavage is observed (E), although micromeres and macromeres are still recognizable. After a few cell divisions, blastomeres form an internal epidermal layer (F) that eventually covers the whole embryo and the external yolk cells after an inverse epibolic movement (G-H). The remaining blastomeres form an embryonic blastema on one side of the embryo, as observed in other neoophoran flatworms (H). In triclads, the formation of a yolk-derived syncytium where disperse cleavage takes place is observed in early embryos (I). Once a certain number of blastomeres is reached, some of them differentiate into two transitory organs (primary epidermis and embryonic pharynx (J), that will be used to ingest the maternally supplied yolk cells (K). After yolk ingestion, the remaining undifferentiated blastomeres proliferate and differentiate into the definitive organs (L), replacing the transitory ones. In all schemes, an idealized cross section of the embryo is represented. In (L), ventral to the bottom and anterior to the left. Yolk cells are colored in light blue, primary hull cells in orange and embryonic cells in gray. Drawings are not to scale. *bl *blastomere, *eb *embryonic blastema, *ec *epidermal cavity, *epi *epidermis, *eph *embryonic pharynx, *epp *embryonic pharynx primordium, *es *eggshell, *pb *polar body, *pep *primary epidermis, *yc *yolk cell, *ys *yolk syncytium.

We have only cursory data about the reproductive biology of these endoparasites (about 20 recognized species [32]), from a study on *Fecampia xanthocephala *and *Fecampia erythrocephala *[77]. The mature, eye-, mouth- and pharynxless adult living in the gut of crustaceans spins a pear-shaped cocoon around itself, loses its gut and lays eggs inside the cocoon. The eggs inside the cocoon have a diameter of about 150 μm and are surrounded by a thin egg shell. Inside each shell, two transparent embryos are embedded in the center of their own colored yolk cell masses. After an early cleavage with no spiral pattern (Figure 6A), the embryos take the form of a horseshoe or an open pouch, defining an internal cavity, which could be considered a kind of gastrulation [77] (Figure 6B). Dispersal of individual blastomeres (*Blastomerenanarchie*) as in prolecithophorans and triclads does not seem to take place. The embryonic pouch closes around a part of the yolk mass, forming a thin embryonic layer (Figure 6C). Large parts of the yolk mass still remain outside the embryo, which subsequently extends its walls to the periphery of the yolk mass, finally incorporating all yolk and attaining a hemispherical shape (Figure 6D). The yolk is gradually absorbed by embryonic blastomeres and becomes restricted to the posterior part of the embryo. Cells in the future anterior end are proliferating most actively, and eventually brain, mouth, pharynx and gut can be recognized, while the ectoderm becomes ciliated and the embryos, cylindrical in shape, start moving. Eventually juveniles hatch, labeled as "larvae". The juveniles possess two eyes, a mouth with an anterior opening, a pharynx and a gut and longer cilia than the adults. After absorbing the remaining yolk in their gut, they enter their new host and mature to adults [27,77]. In a later publication on *Fecampia abyssicola*, freshly hatched juveniles were reported as being eyeless and also lacking a mouth and a pharynx [78], suggesting that Caullery and Mesnil were mistaking "dense bodies" at the anterior end for a mouth and gland ducts for a pharynx.

#### Prolecithophora

Of the circa 200 described species [32] (both marine and freshwater), two species, *Plagiostomum girardi *and *Hydrolimax grisea*, have been the subject of developmental studies [68,79]. In both species, cleavage is unequal and leads to the formation of micromeres and macromeres (Figure 6E). The typical quartet spiral cleavage is not discernible, as the pressure exerted by the large amount of extra-embryonic yolk cells causes blastomeres to detach from each other [79]. This leads to a disperse cleavage (*Blastomerenanarchie*), without regular patterns of divisions. After a few cell divisions, micromeres accumulate in the periphery of the embryo in *Plagiostomum *[68], whereas they become more abundant in the center in *Hydrolimax*, enveloped by a yolk cell mass [79]. In the latter, a cavity in the center of the embryo is formed and lined by future epidermal (ectodermal) cells (Figure 6F). Successively, the embryo shifts towards the surface of the yolk and opens its cavity to the outside, in a process described as inverse epiboly [79] (Figure 6G). During this process, the rest of the blastomeres and yolk cells are incorporated within the embryo by spreading of the epidermis. Organ primordia are formed on the ventral side, as observed in other neoophoran orders (Figure 6H). In *Plagiostomum*, on the other hand, without having undergone inverse epiboly, an embryo with developing organ primordia defines the future ventral side of the animal, from where the definitive epidermis starts spreading around the embryo. Simultaneously, a hull membrane made of yolk cells envelops the dorsal part of the syncytial yolk mass [68]. All prolecithophorans hatch as directly developed juveniles.

#### Tricladida

Together with the Rhabdocoela, the macroscopic Tricladida are the species-richest group of free-living flatworms, and are classified into the mostly cave-dwelling Cavernicola (4 species), the marine Maricola (about 70 species) and the freshwater and terrestrial Continenticola (more than 420 and 820 species, respectively) [32,80,81]. By far, this is the group of flatworms with most available data, not only at the morphological, but also at the molecular level. Although the vast majority of analyzed species are freshwater [82-89], embryological studies cover all the triclad diversity [90,91]. In recent years, the freshwater triclad *Schmidtea polychroa *has become the model species in embryological studies [89,92-97].

Egg capsules contain several small alecithic zygotes together with a large quantity of extra-embryonic yolk cells [82,88,89]. Early stages of development are highly divergent and exclusive for triclads, and thus, are used as apomorphies to define the group. Soon after deposition, yolk cells surrounding the zygote are stimulated to fuse and form a syncytium (Figure 6I). The zygote and early blastomeres do not contribute to this process, and instead become embedded inside the yolk-derived syncytium, where cleavage takes place. As in the Prolecithophora, cleavage does not follow the canonical quartet spiral pattern, but divisions are more or less equal in the Tricladida. In these organisms, the process of disperse cleavage (*Blastomerenanarchie*) is also observed. Since the earliest cell divisions, blastomeres separate from each other and wander around the syncytium, without a regular or discernible pattern.

Once reaching a certain number of cells, a common transient organization to all embryos appears (Figure 6J). Some blastomeres differentiate into a primary (embryonic) epidermis that engulfs part of the yolk syncytium. Both the embryonic epidermis and a layer of yolk cells surrounding the embryonic epidermis, segregating it from other yolk cells, have been labeled "hull membrane" [89]. Other blastomeres migrate towards one pole of the syncytium and differentiate into a temporary pharynx, or embryonic pharynx. The rest of the blastomeres remain in the syncytium in an undifferentiated state, and will be responsible of giving rise to the definitive embryo in subsequent stages. At this developmental point, however, the transient organs allow the embryo to feed on the maternally-supplied extra-embryonic yolk cells, which are swallowed by the embryonic pharynx and gathered in the center of the embryo (Figure 6K). While the earliest observers of triclad development tended to compare the formation of this transient yolk-feeding embryo with gastrulation and germ layer segregation [82-84], the most widely accepted view states the absence of true gastrulation movements in triclads [87]. However, recent molecular studies have demonstrated the expression of evolutionary conserved gastrulation-related genes during these early processes, such as *snail, twist, foxA *and *β-catenin *[96], which suggests that although in a very divergent way, ancient mechanisms of early cell fate specification and embryonic patterning are still present in triclad embryos.

After yolk ingestion, the remaining undifferentiated blastomeres (expressing stem cell associated gene markers, such as *vasa *and *tudor *[94]) proliferate and differentiate into the definitive organs, which replace the transient early-developed pharynx and epidermis (Figure 6L). This process was usually described to involve the formation of three main ventral anlagen (an anterior brain primordium, a central pharynx primordium, and a posterior or caudal primordium) as in other neoophoran flatworms [98]. However, recent studies on *S. polychroa *demonstrate that the appearance of the definitive cell types, tissues, and organs occurs much more diffusely [89,97].

Finally, the establishment of the embryonic polarity has been a matter of debate since the early descriptions of triclad embryogenesis. There has been a trend towards assuming that the point in the yolk-derived syncytium where the embryonic pharynx develops, already corresponds to a pole of the future definitive embryo, often the ventro-posterior region. However, the analysis of the molecular mechanisms controlling axial polarity in adult triclads, namely the canonical Wnt pathway and the BMP pathway, has demonstrated that these become active only after the yolk has been ingested and the undifferentiated blastomeres start to differentiate into the adult cell types [96]. How the early transient embryo is patterned thus remains as a major question in triclad embryological studies. From a single cocoon, more than a dozen juveniles can emerge [99].

#### New answers to old questions

The presence of more reliable phylogenies of the phylum Platyhelminthes offers a unique opportunity to place the above described developmental modes (summarized in Table 1) under a more coherent evolutionary framework, and thus, shed light on the origin and diversification of this group of animals. In the following section, we discuss the most informative embryonic characters and put forward evolutionary hypotheses that can be useful for future developmental studies, in particular concerning spiral cleavage in the presence of extraembryonic yolk cells, gastrulation events, axis formation in the embryo and indirect development.

##### Spiral cleavage and ectolecithic development

Current phylogeny supports considering quartet spiral cleavage as the ancestral developmental mode in Platyhelminthes [100]. The presumably basal position of macrostomorphs to the whole group of rhabditophorans (Figure 1) implies that the loss of a spiral pattern after the first three cleavage divisions observed in this lineage is independent from the loss observed in ectolecithic flatworms, especially considering that the ectolecithic lecithoepitheliates show a spiral cleavage pattern until gastrulation [29]. In a similar way, the developmental deviations found in polyclads - such as the degeneration of fourth quartet macromeres and of micromeres 4a-c - are probably apomorphies of this group, since they are not present in those taxa of neoophoran platyhelminthes that retain a quartet spiral cleavage [26,29]. The shift of the mesentoblast in polyclads from 4d to 4d^2 ^can either be considered a polyclad apomorphy [51], a view supported by the presence of a 4d mesentoblast in lecithoepitheliates [29], or it can be a plesiomorphic state for all spiralians [57]. Further study of the embryonic development in the Catenulida may be instructive with regard to the plesiomorphic state of cleavage patterns in the Platyhelminthes.

Concerning cleavage in neoophoran groups, it is important to note that the presence of external yolk cells does not necessarily imply the loss of spiral cleavage, as exemplified in lecithoepitheliates and proseriates [28,29]. In fact, the complete absence of spiral cleavage is a shared trait for the clade Neodermata-Rhabdocoela-Adiaphanida (the Eulecithophora *sensu *de Beauchamp [101]) and also by the Bothrioplanida, which might thus be explained by a single evolutionary event at the base of this group. However, there are significant differences between cleavage in rhabdocoels and adiaphanids (e.g. first equatorial division and formation of a compact morula in rhabdocoels; disperse cleavage in prolecithophorans and triclads), and therefore, they are likely independent adaptations to their ectolecithic condition.

According to some descriptions, small abortive blastomeres with compacted chromatin and little cytoplasm are occasionally formed in proseriates [26,28]. These blastomeres remain in the periphery of the embryo or are eventually included in the yolk mass. Their fate is not clear: some of them degenerate, while others seem to participate in the formation of the body wall epithelium. Although some authors compared this situation with the one observed in triclads and prolecithophorans [30] and thereby considered it as a precursory stage in the evolution of neoophorans, we believe more data is necessary to confirm these similarities.

The current relationship between Prolecithophora and Tricladida based on molecular data [25] leads to grouping two taxa with disperse cleavage within the same clade. The description of the embryonic development of the Fecampiida does not warrant an interpretation that disperse cleavage takes place in this group [77]. Still, a common origin to this divergent mode of cleavage can be proposed for the Prolecithophora and the Tricladida. Nonetheless, there are also differences between these two groups: formation of micromeres and macromeres in prolecithophorans, like in the ancestral mode [68,79]; cleavage within a yolk-derived syncytium in triclads. For the Bothrioplanida, disperse cleavage was described just like in prolecithophorans and triclads. Given their current, but uncertain, position in the phylogenetic tree (a closer relationship of the Bothroplanida with the Prolecithophora and Tricladida seems possible), disperse cleavage has either emerged independently in *Bothrioplana *on the one hand and prolecithophorans and triclads on the other hand, or less likely, a stereotypic cleavage pattern has independently emerged anew in rhabdocoels, fecampiids and neodermatans. To this respect, in a recent manuscript [102], Azimzadeh and coworkers demonstrated the absence of centrosomes in the triclad *S. mediterranea *(as well as in the neodermatan *Schistosoma mansoni*) and suggested that this loss occurred concomitantly with the loss of spiral cleavage (and the emergence of disperse cleavage) in the ancestor of triclads and schistosomes. Although appealing, from the points discussed above one can conclude that this hypothesis requires studying the absence or presence of centrosomes in other groups of flatworms, in particular bothrioplanids, rhabdocoels, prolecithophorans, fecampiids and the rest of the neodermatans, to gain actual evolutionary significance.

A different view of the evolution of the cleavage pattern was proposed by Bogomolov [35,44] after studying the embryonic development of a number of turbellarians, from catenulids to macrostomids and rhabdocoels. Most strikingly, his accounts of spiral cleavage in *Macrostomum viride *are in stark contrast to observations of hull cell formation in the same genus. Also in several rhabdocoels, he described early cleavage as following a spiral pattern, a view not supported by most other authors. Finally, in catenulids, only Bogomolov gives an account of spiral cleavage until at least the 16-cell stage, again a singular observation that stands and falls with this author. Most of the species examined by Bogomolov have not been subjected again to embryonic studies, and so it remains an unresolved challenge to determine how conserved spiral cleavage is in several turbellarian taxa.

Finally, how did the changes in the ancestral quartet spiral cleavage affect cell fates during early embryogenesis? Polyclads have been shown to follow a determinative mode of cleavage, in that the loss of blastomeres during early development cannot be compensated by the remaining blastomeres [55]. Due to experimental difficulties, ablation experiments are still lacking in the neoophorans, and thus it remains unclear whether seemingly irregular cleavage patterns labeled as disperse cleavage are still determinative or whether they are possibly of the regulative mode [26]. Interestingly, some peculiar features of rhabditophoran flatworms, such as the lack of proliferating cells in the epidermis, are shared at least by macrostomorphans, polyclads and triclads [8], indicating a similar general fate of blastomeres or germ layers in archoophorans and neoophorans.

##### Gastrulation

Gastrulation can be defined as the series of highly coordinated cell and tissue movements that lead to the formation of an embryo with distinct cell layers (ectoderm-endomesoderm) and with a basic body plan [103]. The mechanisms involved are numerous and diverse, even within the same phylum, as is the case for instance in cnidarians [104]. The ancestral mode of gastrulation for platyhelminths seems to be an epibolic movement of the animal micromeres over the vegetal-most blastomeres, as seen in polyclads and lecithoepitheliates and other phyla with quartet spiral cleavage. The amount of yolk decisively influences the mode of gastrulation [105]. The formation of a blastocoel is apparently not related to ento- or ectolecithy, as both the entolecithal polyclads and some ectolecithal lecithoepitheliates lack a blastocoel (stereoblastula), while proseriates form a coeloblastula [28,29]. However, the presence of external yolk cells seems to have a remarkable effect on the gastrulation of ectolecithic flatworms. Different strategies to incorporate part of, if not all, the external yolk into the embryo have been described. The Lecithoepitheliata and Proseriata still show a recognizable epibolic gastrulation, although the movement of the animal micromeres over the vegetal macromeres also engulfs part of the yolk mass [26,28,29]. On the contrary, the Rhabdocoela and the Adiaphanida present divergent modes of gastrulation, in which blastomere movements are less archetypal.

The gastrulation proposed for the order Prolecithophora [79] has some similarities with the formation of the primary epidermis in triclads, which supports considering this stage of development of triclads as a divergent gastrulation. However, there is no embryonic pharynx in prolecithophorans, which may be related to the fact that gastrulation incorporates all the maternally-supplied yolk cells within the embryo. The remaining adiaphanids, the fecampiids, first only encompass part of the yolk mass with embryonic blastomeres, which subsequently extend to envelop all yolk [77].

Associated with gastrulation and incorporation of the yolk cells, the term hull membrane (or *huell *membrane) has been often used. Nevertheless, it has been applied to tissues and organs of different embryonic origin, and is thus unlikely to be homologous. In macrostomids, it refers to the yolk mantle surrounding the embryo derived from the macromeres 2A-2D [38]. In lecithoepitheliates and proseriates, the transient epidermis made of 2nd and 3rd quartet micromeres that engulfs the yolk cells is also called a hull membrane [29]. Similarly, unspecified embryonic blastomeres in bothrioplanids and yolk cells in some prolecithophorans, form a transitory layer called hull membrane [26,68,79]. In triclads, the term has confusingly been used to designate both the embryonic epidermis made from blastomeres, and the yolk cells that do not contribute to the yolk-derived syncytium but that remain in the periphery of the embryo and isolate it from the rest of the embryos of the same egg capsule [89]. Finally, the thin - sometimes yolk-derived, sometimes of embryonic origin - epithelium present in rhabdocoels has been also named hull membrane [67], whereas in the proseriate *Monocelis fusca *a layer made of yolk cells around the yolk mass was labeled vitellocyte epithelium [28]. This ubiquitous use of the term hull membrane seems to suggest a common evolutionary origin for these structures, which is probably not the case.

**Figure 7:**
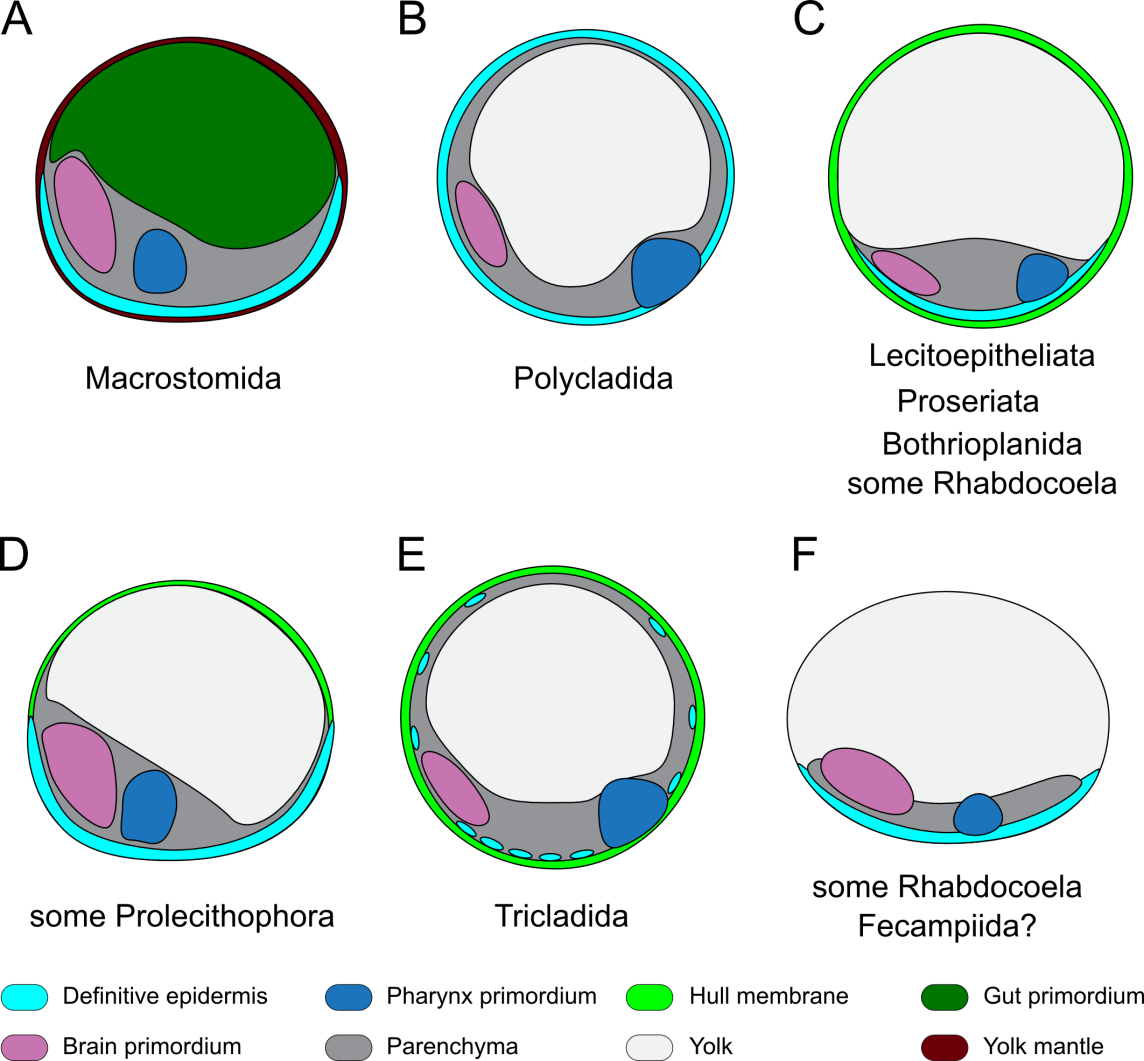
**The phylotypic stage in free-living flatworms**. (A-F), schematic representations of embryos of the major groups of free-living flatworms at the moment of organ specification (stage 6 according to [67]). Regardless of their particular early steps of embryogenesis, free-living flatworm embryos exhibit the greatest similarity at this point of development, being also the stage at which the basic body plan is defined. In all groups, an anterior neuropile and a mid-posterior ventral pharynx start to differentiate, whereas the abundant yolk (either external or internal) adopts a dorsal position. In macrostomids and neoophoran groups, the definitive epidermis differentiates from the ventral side of the embryo to the most dorsal regions, superseding the yolk mantle or the hull membranes, respectively. These similarities led some authors to propose this time point as the phylotypic stage of Platyhelminthes [75], a concept that we extend in this work to other groups of free-living flatworms not previously considered. In all schemes, anterior is to the left and ventral to the bottom. Drawings are not to scale.

Regardless of the term used, a common feature observed in most of the studied ectolecithic platyhelminths is the formation of a transitory epidermis (in some cases even successive transitory epidermises) to engulf part of the yolk cells during the early stages of development. In the entolecithic *Macrostomum*, a yolk mantle is formed around the embryo (Figures 2D, 7A), possibly accomplishing a similar goal [43]. Also the primary epidermis of bothrioplanids, some prolecithophorans and triclads can be considered under this definition, although its formation follows a different sequence of events than in lecithoepitheliates and proseriates due to the presence of disperse cleavage. The archoophoran polyclads and some neoophoran prolecithophorans, rhabdocoels and the endoparasitic fecampiids are the only turbellarians without this transitory epidermis, which is later being replaced by the definitive epidermis. Based on the function and the origin from embryonic blastomeres (macromeres or micromeres), a common origin for the transitory epidermis(es) found in many ectolecithic platyhelminths (and possibly also in *Macrostomum*) can be hypothesized. This trait may have been lost in fecampiids and some prolecithophorans and rhabdocoels. In some of the latter an analogous structure would have arisen, but from yolk cells, as in some proseriates [28], some prolecithophorans [68] and triclads [89]. Ax [3] proposed a classification of neoophoran development based on the different ways of enveloping the yolk cells: complete (type 1) or partially (type 2), or with a "yolk larva" (triclads, type 3) or by phagocytosis (type 4). However, more studies, in particular with molecular markers, are necessary to demonstrate these affinities.

What is the evolutionary advantage of extra-embryonic yolk cells? In neoophoran flatworms, often several oocytes are contained in one eggshell, which are competing for the yolk resources. If an embryo has abortive development in an early to mid stage, its resources can be taken up by its siblings. Another way of dealing with the tissue of died embryos in a cocoon is seen in the polyclad *Planocera reticulata*, where embryos just after the formation of mouth, gut and a ciliated epidermis are capable of feeding on the remnants of their dissociated siblings, leading to Kato's larvae which are comparatively big when hatching [52].

##### Embryonic polarity

Simultaneously with the appearance of ectolecithic development, changes in the specification of the embryonic polarity occurred. While in macrostomids the AV axis represents the future AP axis of the embryo [41], as in many other spiralians, in polyclads a shift of the AV axis by 90° to the AP axis occurs [45]. In some groups of neoophoran platyhelminths, in particular lecithoepitheliates and proseriates, the AV axis corresponds to the definitive DV axis, where the animal pole becomes the ventral side. In prolecithophorans and triclads, as a consequence of the disperse cleavage, this correlation is not so clear and the establishment of the definitive polarity seems to be a late developmental event, at least in triclads [96]. Some authors have explained this change in the specification of the embryonic polarity in the transition from basal groups to neoophoran platyhelminthes by a 90° shift in the location of maternal determinants [30]. Nevertheless, the lack of basal embryological studies in most of these groups makes it difficult to propose a hypothesis. The study of conserved molecular pathways involved in the establishment of the axial identities in other metazoans can be a fruitful approach to gain new insights into this important question.

##### Indirect development

Indirect development has been described in three groups of free-living platyhelminths: catenulids with Luther's larva, polyclads with Müller's, Goette's and Kato's larva, and fecampiids. In catenulids, Luther's larva was only observed for a single species of the genus *Rhynchoscolex, R. simplex *[36], while all other described catenulid juveniles are direct developers. The only "larval" features of Luther's larva are the presence of a statocyst in the larva, which is lost during post-embryonic development, and a band of longer cilia in the head region. However, other species of *Rhynchoscolex*, e.g. *R. diplolithicus*, as well as many other catenulids, do retain a statocyst as adults [36], so the presence of a statocyst cannot be considered a larval character. It therefore seems hardly justifiable to call the juvenile of *R. simplex *a larva, and we propose to consider Luther's larva being a directly developing juvenile. A similar argument can be made for the larva of fecampiids, where also the adults undergo a modification (reduction), while the juveniles represent the unmodified stage [77], although later observations suggest that already the hatching juveniles are devoid of eyes, mouth and pharynx [78]. However, these findings were based on unfixed material from research ships, and histology of this material did not give enough detail ascertaining the absence of these organs [78].

Polyclad larvae, on the other hand, do possess unique features not found in the adult worm, most notably their lobes, which are eventually resorbed during metamorphosis [48,53,60,62] (with one exception, *Graffizoon lobatum*, which is considered a neotenic Müller's larva [106]). In the spherical Müller's and Goette's larvae, but not in the already flattened Kato's larva, also a dorsoventral flattening takes place during metamorphosis, and the number of eyes increases [62]. Still, most parts of the larval body are retained for postembryonic development, similar to e.g. the annelid *Platynereis dumerilii*, where the trochophore larva undergoes only little metamorphosis, but is becoming posteriorly segmented and elongates as the worm grows [107].

The relationship of Müller's, Goette's and Kato's larva and direct developing juveniles (Figure 3) in polyclads has not been resolved. In some acotylean genera, two or three types of development can occur. For example, *Planocera reticulata *develops as a Kato's larva, while *Planocera multitentaculata *features a Müller's larva [53] and *Planocera elliptica *a Goette's larva [48,108]; *Hoploplana inquilina *hatches as a Müller's larva [51], whereas *Hoploplana villosa *is a direct developer [52]; in *Stylochus*, Müller's and Goette's larvae were described for several species, as well as direct developers [109]. All three larval types are united by the presence of lobes adorned with long cilia, but the number of lobes - from four in Goette's larvae up to ten in some late Müller's larvae [110] - and also the shape of the larvae's bodies - spherical in Müller's and Goette's larvae, dorsoventrally flattened in Kato's larva - varies.

In addition, some authors have proposed the presence of a "cryptic larva" in triclads [93,111], based on the presence of specific organs in the early yolk-feeding embryo (embryonic pharynx and primary epidermis, mainly) that are later on superseded by the definitive organs of the hatchling. This morphological independence of the early triclad embryo seems to be supported by the existence of two clearly different molecular profiles between these two main stages of triclad embryos [96]. Nevertheless, the current phylogeny supports the development of a yolk-feeding embryo as a triclad apomorphy, as it is not shared by any of the closely related groups. Accordingly, the possibility that the transient yolk-feeding embryo of triclads is homologous (i.e. share a common origin) to the polyclad larvae, as proposed by [93], seems unlikely.

Finally, all neodermatan flatworms have one or more larval stages, but these larvae are probably adaptations to their parasitic life style and not homologous to the polyclads' larvae [2,112].

Was a larva ancestral to all Platyhelminthes? It seems most parsimonious to consider the maximal indirect development (*sensu *[113]) observed in polyclads as an autapomorphy of this group, with the ancestral platyhelminth being a direct developer. This is also supported by the presence of strict direct development in the phylum Gastrotricha, one of the proposed sister taxa of the Platyhelminthes [10]. If, on the other hand, the polyclads' larvae were a plesiomorphic character for the Platyhelminthes, according to the current phylogenetic tree (Figure 1) this would imply the loss of such a larva in the Catenulida, the Macrostomorpha, some acotylean Polycladida and the Neoophora. In such a case, a possible homology of the larval forms in polyclads and the trochophore larva of the Trochozoa could be explored [27,112]. At least for polyclads, a larva similar to Müller's larva was probably present in the stem species of polyclads, because it is present in both Cotylea and Acotylea [62].

##### Phylotypic stage

From the material presented above, one can deduce that early development among the different groups of platyhelminths is very variable. Not only is the organization of the oocyte (entolecithic versus ectolecithic) different, but also the strategy followed by each group to deal with increasing amounts of yolk. Therefore the question arises, when and how is the basic body plan of a platyhelminth established?

Universally, for all neoophorans and also *Macrostomum*, the embryonic primordia divided into brain, pharynx and genital apparatus, are being formed at the ventral side of the embryo, from which the epidermis is starting to encompass yolk cells or yolk-rich blastomeres being situated at the dorsal side [3,30]. The latter process bears similarity to the epibolic gastrulation in polyclads [30,71]. Some authors have applied the concept of phylotypic stage to the study of flatworm embryology [75]. Based on their descriptions of the embryonic development of polyclads and rhabdocoels [45,67,74], stage 6 (according to the staging proposed by [67]) was suggested as the moment in development when the basic features of the body plan of a (rhabditophoran) platyhelminth are defined, namely a ventral definitive epidermis, an anterior neuropile with incipient ventral nerve cords, a ventral pharynx and developing muscle layers, and a pair of lateral bands with nephridial cells and myoblasts, which closely corresponds to the types proposed by earlier authors [3,30]. The most recent studies of the embryonic development of triclads support considering stage 6 as the point in development when the basic body plan of a triclad is defined, as in other platyhelminths [89,96,97]. Interestingly, at the same time, triclads start being able to regenerate [114], suggesting that developed organ systems (e.g. nervous system, stem cell system) are a necessary prerequisite for regeneration in flatworms [115].

Figure 7 illustrates the basic structure of the embryos of the major free-living flatworm groups at this developmental time point. As it can be observed, all of them present equivalent regions, and despite small morphological differences, the overall anatomy is very similar. Nevertheless, it is again necessary to further investigate the embryonic development of most of the flatworm groups, in particular at the molecular level, to validate this hypothesis and enhance our understanding of the evolution of the body plan of this diverse phylum.

## Conclusions

In this manuscript, we exhaustively review the embryogenesis of the free-living Platyhelminthes *sensu stricto*, and discuss the classical knowledge and the recent advances under the most up-to-date consensus phylogenetic tree. The last comparative work on the development of flatworms dates back to 1990 [31]. Since then, significant changes have occurred in the position of Platyhelminthes within the animal tree of life and the groups that comprise this phylum, as well as in the way flatworm embryogenesis is studied.

Great differences are observed during early development among the different groups of Platyhelminthes. Entolecithal eggs, a quartet spiral cleavage pattern, epibolic gastrulation and a determinative development are most likely ancestral for the early embryogenesis of Platyhelminthes, although significant divergences are found in macrostomids and polyclads, the two best known basal groups of flatworms. In macrostomids, embryonic hull cells enveloping the embryo seem to foreshadow the hull membrane in neoophorans, whereas polyclads do not show any kind of hull formation. However, three peculiarities, deviating from most other spiralians, are found in polyclads: the complete endoderm is formed by 4d, the mesentoblast is 4d^2^, not 4d, and the macromeres after the sixth cleavage division are smaller than the micromeres and do not contribute to any embryonic tissue formation. The understudied Catenulida, as the sister group to the Rhabditophora, emerges as a key taxon to gain deeper understanding of the origin and radiation of this phylum.

The appearance of the ectolecithal egg seems to have occurred once in the evolutionary history of Platyhelminthes. Although early branching neoophoran flatworms (i.e. lecithoepitheliates and proseriates) still show quartet spiral cleavage, more divergent groups have evolved new and characteristic strategies (irregular cleavage in rhabdocoels, disperse cleavage in bothrioplanids, prolecithophorans and triclads). Simultaneously, gastrulation has been severely modified. While in lecithoepitheliates (and to some extent also in proseriates) an epibolic migration of the animal micromeres over the vegetal macromeres is recognizable, the segregation of the definitive germ layers and specification of cell fates do not involve prototypic cell movements in the other neoophoran groups. Related to gastrulation, the differentiation of some blastomeres into a transitory epidermis (hull membrane) to incorporate part, if not all, the maternally supplied yolk cells into the embryo seems to be an ancestral character of neoophoran flatworms. Nevertheless, this trait has undergone great variation in some groups (such as bothrioplanids, some prolecithophorans and triclads) and might have been even lost in others (i.e. fecampiids and some prolecithophorans and rhabdocoels).

Regardless of their early development, all free-living Platyhelminthes seem to proceed through a developmental stage in which the basic common body plan is established. Although most of the platyhelminths develop from this stage directly to the adult form, there are some species of polyclads with an intermediate true larval form (Müller's, Goette's and Kato's larva). The phylogenetic position of polyclads within Platyhelminthes suggests that indirect development is not ancestral, although homology between polyclad larvae and the trochophora larva has been proposed [112]. Regeneration capacity of embryos is established only after reaching the phylotypic stage, which so far was shown for triclads only [114]. Data from other flatworm groups are lacking, but similar results are expected.

With this manuscript we aim to review and highlight the great diversity, novelties and appealing commonalities observed during embryogenesis in the different groups of free-living Platyhelminthes. All these data, together with the position of Platyhelminthes within spiralians, make the study of flatworm embryology one of the most exciting research fields in modern comparative evolutionary biology. In this sense, the application of the most recent molecular approaches to some key groups will significantly improve our understanding of metazoan evolution.

## Competing interests

The authors declare that they have no competing interests.

## Authors' contributions

JMM-D and BE designed the study, created the fiures, critically analyzed the bibliography and wrote the manuscript. Both authors read and approved the final manuscript.
